# Lessons from Exploring Chemical Space and Chemical Diversity of Propolis Components

**DOI:** 10.3390/ijms21144988

**Published:** 2020-07-15

**Authors:** Trong D. Tran, Steven M. Ogbourne, Peter R. Brooks, Norberto Sánchez-Cruz, José L. Medina-Franco, Ronald J. Quinn

**Affiliations:** 1 GeneCology Research Centre, School of Science and Engineering, University of the Sunshine Coast, Maroochydore DC, Queensland 4558, Australia; sogbourn@usc.edu.au (S.M.O.); PBrooks@usc.edu.au (P.R.B.); 2 Department of Pharmacy, School of Chemistry, Universidad Nacional Autónoma de México, Mexico City 04510, Mexico; norberto.sc90@gmail.com (N.S.-C.); medinajl@unam.mx (J.L.M.-F.); 3 Griffith Institute for Drug Discovery, Griffith University, Brisbane 4111, Australia; r.quinn@griffith.edu.au

**Keywords:** honey bee propolis, stingless bee propolis, natural products, phenolics, terpenoids, chemoinformatics, chemical space, chemical diversity

## Abstract

Propolis is a natural resinous material produced by bees and has been used in folk medicines since ancient times. Due to it possessing a broad spectrum of biological activities, it has gained significant scientific and commercial interest over the last two decades. As a result of searching 122 publications reported up to the end of 2019, we assembled a unique compound database consisting of 578 components isolated from both honey bee propolis and stingless bee propolis, and analyzed the chemical space and chemical diversity of these compounds. The results demonstrated that both honey bee propolis and stingless bee propolis are valuable sources for pharmaceutical and nutraceutical development.

## 1. Introduction

The emergence of new infectious and chronic diseases makes the need for new drugs paramount [1]. Although the search for new drugs can begin from different sources, natural products have proven to be one of the richest sources of bioactive ingredients and molecules with privileged scaffolds for the discovery and development of new and novel drugs [2,3,4,5,6]. They were historically the sources of all folk medicines [7]. Having evolved over millions of years, structures of natural products have been fine-tuned by nature for optimal bioactivity [5]. Modern studies revealed natural products possess an advantageous structural foundation and cover a wide range of biologically relevant chemical space that cannot be efficiently explored by synthetic compounds [8,9,10]. These features positively influence the probability of the clinical success of natural product-based drug candidates [11]. A detailed analysis of 1394 new small molecule drugs approved by the US Food and Drug Administration (FDA) between 1981 and 2019 [6] revealed that 32% of those drugs were natural products or direct derivatives of natural products.

Propolis, which is a product of bees, has been used in the folk medicine of many cultures to treat microbial infections since the year 300 B.C. [12]. The name “propolis” originally came from the Greek words meaning “defence of the city” (“pro” meaning “to defend” and “polis” meaning the city) [13]. Historically, the Greeks and the Romans used propolis for treating bruises and suppurating sores; the Egyptians applied propolis for embalming cadavers and preventing infections; the Arabians utilised propolis as an antiseptic, a wound healing agent, and a mouth disinfectant; the Incas described propolis as an antipyretic agent [14]. Owing to its antibacterial characteristics, propolis was approved as an official drug in the London pharmacopoeia in the 17th century and, since then, has become more popular [15]. Propolis was also used to treat wounds during World War II (1939–1945) [14]. In 1969, propolis was approved as human and veterinary drugs with several applications, including the treatment of tuberculosis in the Union of Soviet Socialist Republics [14].

**Figure 1:**
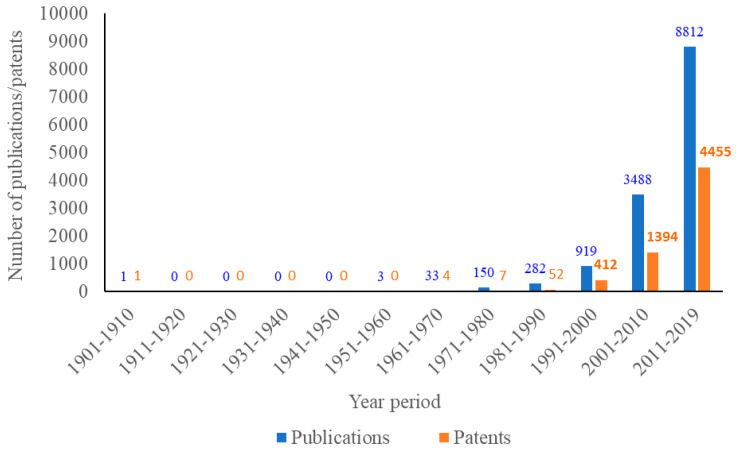
Number of scientific outputs containing the word “propolis” per decade (publications include books, clinical trials, commentaries, conferences, dissertations, editorials, journals, letters, patents, preprints, reports, and reviews—searched on SciFinder database (Chemical Abstract Service) on 2nd Jan 2020).

Since the early 21st century, there has been a significant increase in scientific publications on propolis (Figure 1). Studies validated the antimicrobial property of propolis extracts and discovered additional therapeutic properties, including antioxidant, anti-inflammatory, antidiabetic, dermatoprotective, antiallergic, laxative, immunomodulatory, and anticancer activities [16]. Nowadays, propolis is used in pharmaceutical and cosmetic industries as a unique natural constituent in cough syrups, dietary supplement tablets, antiacne creams, facial and body creams, ointments, lotions, toothpastes, and mouthwash products [17]. It has also been used in some foods and beverages as an alternate preservative agent or food supplement [13]. The first patent referring to propolis was described in 1904 with a claim of using propolis as one of the compositions to treat piano pins and strings [18]. Propolis-related patents numbered about 500 by the end of the 20th century and increased dramatically by almost three-fold and nine-fold in the first and second decades of the 21st century, respectively. The number of patents referring to propolis from 2011 to 2019 accounted for 50% of its total publications in the same period (Figure 1). Medicinal and nutraceutical products were observed in high frequency in these patent applications.

Over the last two decades, the relationships between the pharmacological properties of propolis and its components have attracted the attention of the scientific community. It is known that raw propolis, in general, consists of about 50% resin, 30% wax, 10% essential oils, 5% pollen, and 5% others (including amino acids, peptides, dead bees, and soil) [19]. By employing different chromatography and spectroscopic techniques, such as thin layer chromatography, gas chromatography (GC), high-performance liquid chromatography (HPLC), mass spectroscopy (MS), and nuclear magnetic resonance spectroscopy (NMR), over 300 volatile and non-volatile components have been identified in propolis [20]. Among them, phenolics and terpenoids have been confirmed to play important roles in the biological activities of propolis [17,21,22,23].

Several comprehensive reviews have reported the natural compositions found in propolis [13,17,20,24,25,26,27,28,29,30] and their biological activities [13,14,16,22,23,24,31,32,33,34,35]. However, the chemical space and the chemical diversity of propolis components have been underexplored. In this article, we review all compounds isolated from both honey bee propolis (HBP) and stingless bee propolis (SBP), which have been fully characterized and reported in the literature up to the end of 2019. Compounds identified from GC-MS and LC-MS were excluded in this study. As a result of the search, we assembled a database with 578 unique compounds. The chemical space and chemical diversity of the propolis components were characterized to assess their potential for future developments as pharmaceuticals and nutraceuticals.

## 2. Propolis Components: Chemistry and Geographical Distributions

### 2.1. Propolis Classification

#### 2.1.1. Honey Bee Propolis

The honey bee genus *Apis* is the only genus of the tribe Apini in the Apidae family [36]. This genus consists of 11 species, including *A. andreniformis*, *A. binghami*, *A. breviligula*, *A. cerana*, *A. dorsata*, *A. florea*, *A. koschevnikovi*, *A. laboriosa*, *A. mellifera*, *A. nigrocincta*, and *A. nuluensis* [36]. These bees are well known for their production of honey, as well as being the pollinator of the majority of the worlds commercial fruit crops [36]. *Apis mellifera*, which is the most common species of honey bee, is indigenous to Europe, Africa, and the Middle East, but nowadays has been found in almost all regions of the world [28]. It has been known that *A. mellifera* produces a high yield of propolis, while other honey bee species provide relatively small or no propolis [21,36].

Honey bee propolis (HBP) is produced mainly from the exudates of plant tissues, such as flower buds, bark and fruit, mixed with saliva and beeswax by bees [24]. The bees gather plant exudates, often referred to as resin, which contain substances involving chemical defense systems to protect plants against their herbivores, bacteria, fungi, moulds and viruses, during the warm part of the day when resin is soft [36]. The bees pack resins on their hind legs and transport them back to the hive to fill hive cracks, reducing the size of the hive entrance to prevent the invasion of other insects and to seal up the inside of the hive by mixing it with wax to maintain an antiseptic environment for the colony and larvae [37,38]. Physically, propolis is soft, pliable, and very sticky when warm, but becomes hard and brittle when cold. Its melting point is around 65 °C, but in some samples it is as high as 100 °C [17]. It has a pleasant aromatic smell and varies in colour depending on its plant sources and age [24]. On average, one bee can bring 10 mg propolis per flight to its hive, and one colony collects about 50–150 g propolis annually [39]. With the application of specialised collection procedures, the sub-species of the European honey bee, *A. mellifera causasica*, can produce 250–1000 g of propolis annually, per hive [21,40].

#### 2.1.2. Stingless Bee Propolis (Cerumen or Geopropolis)

Stingless bees belonging to the tribe Meliponini, in the Apidae family, are the largest group of eusocial bees on Earth, and are closely related to the common honey bee, *A. mellifera* [41]. About 619 stingless bee species in 61 genera have been found in tropical regions of America (South and Central Americas), Africa, Southeast Asia, and Northern Oceania [41]. It is estimated that 40–90% of native or cultivated plant species in the tropics are pollinated by stingless bees [33]. Compared to honey bees, stingless bees have many different features, including colony size, nesting biology, brood comb composure, bee queen production, stocking strategy, and bee recruitment mechanisms [41]. The most significant difference is that they are ‘stingless’, which refers to the fact that their sting is highly reduced, and they do not use it for defense. Instead, some stingless bees develop other methods to protect themselves, such as a strong bite or increasing the pain of the bite by producing formic acid through their mandibular glands [29].

**Figure 2:**
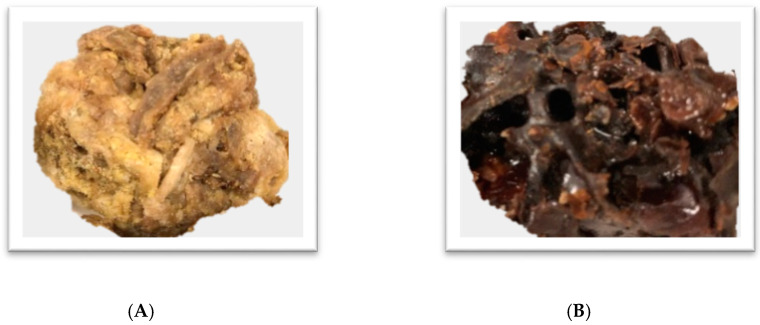
Propolis of the honey bee *A. mellifera* (**A**) and the Australian stingless bee *Tetragonula carbonaria* (**B**).

Both honey bees and stingless bees are able to produce propolis (Figure 2). While the honey bee’s nests are structurally double-sided hexagonal combs built primarily from wax and their hives are sealed by propolis resin, the nests of stingless bees are more complex with a great variety of forms and size, and are made primarily from a propolis-based substance called cerumen [42]. The terms cerumen and propolis are used interchangeably in the literature with respect to stingless bees. Propolis from stingless bees is sometimes found as a mixture of resin and clay or soil. Therefore, this product is also called geopropolis [29].

### 2.2. Chemical Components of Propolis

**Figure 3:**
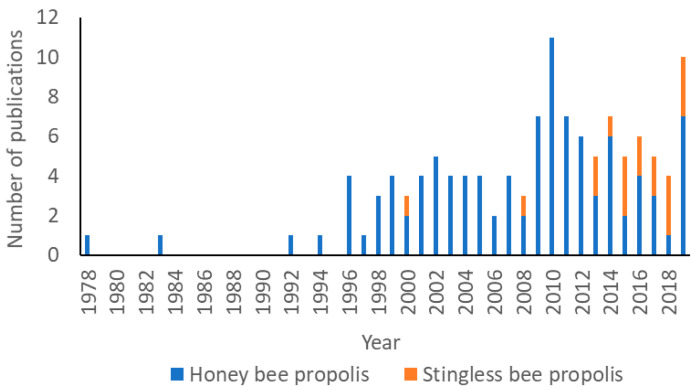
Publications reporting compounds discovered from propolis (*n* = 122) [25,42,43,44,45,46,47,48,49,50,51,52,53,54,55,56,57,58,59,60,61,62,63,64,65,66,67,68,69,70,71,72,73,74,75,76,77,78,79,80,81,82,83,84,85,86,87,88,89,90,91,92,93,94,95,96,97,98,99,100,101,102,103,104,105,106,107,108,109,110,111,112,113,114,115,116,117,118,119,120,121,122,123,124,125,126,127,128,129,130,131,132,133,134,135,136,137,138,139,140,141,142,143,144,145,146,147,148,149,150,151,152,153,154,155,156,157,158,159,160,161,162].

**Figure 4:**
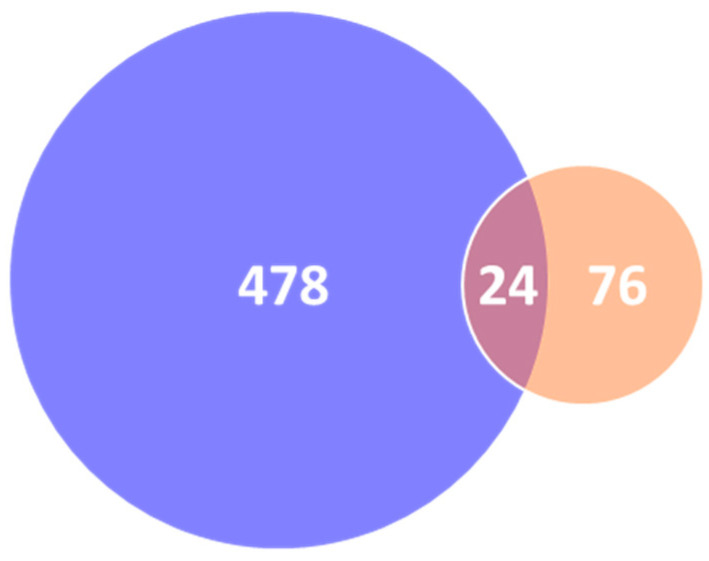
Number of compounds isolated from propolis (*n* = 578) (Blue: HBP (*n* = 502); orange: SBP (*n* = 100)) (overlapped compounds were removed).

**Figure 5:**
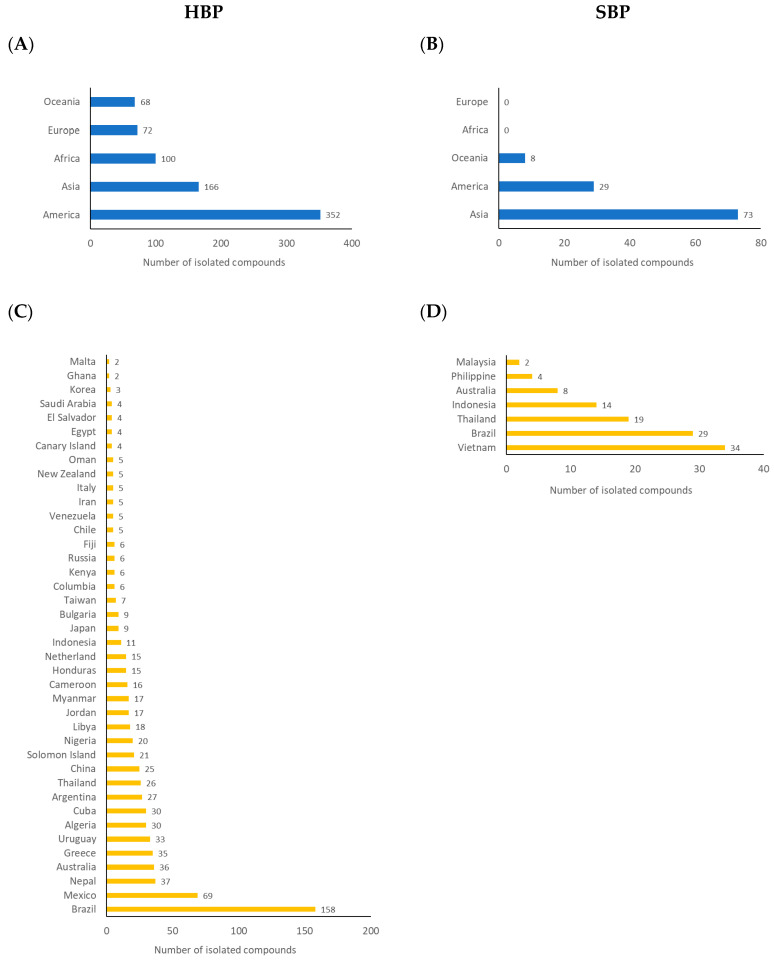
Geographic distribution of compounds isolated from HBP (**A**,**C**) and SBP (**B**,**D**) based on continents (**A**,**B**) and countries (**C**,**D**).

Chemical investigations of HBP have been undertaken since the mid-20th century. However, the literature reports of the discovery of HBP compositions were relatively small prior to 1996, with a significant increase since 2010 (Figure 3). Potentially, this increase in interest was stimulated by the scientific validation of the pharmacological properties of HBP during the late 1990s and early 2000s [30]. Up to December 2019, there were 502 different natural products isolated and characterised from materials collected in 40 countries (Figure 4 and Figure 5C, and Supporting Information 2). In contrast, propolis produced by stingless bees has only relatively recently been studied with the first isolation of three diterpenes from the Brazilian *Melipona quadrifasciata anthidioides* SBP in 2000 [43]. In the early 2000s, most studies were dedicated to Brazilian SBP. However, more recently the number of publications on SBP from Southeast Asia and Australia has grown significantly. A total of 100 compounds have been identified from SBP from 2000 to 2019 (Figure 4, and Supporting Information 2). A total of 24 of the 100 compounds have been previously identified in HBP.

America, particularly Central and South America, is a continent where the most HBP compounds (352 compounds) have been identified and reported, followed by Asia (166 compounds), Africa (100 compounds), Europe (72 compounds), and Oceania (68 compounds) (Figure 5A). Among the 40 countries where compounds have been isolated and identified from HBP, Brazil is a leader with 158 compounds discovered, followed by Mexico (69 compounds), Nepal (37 compounds), Australia (36 compounds), and Greece (35 compounds) (Figure 5C).

In term of SBP, most compounds have been reported from Asian SBP (Figure 5B). Only seven countries, including Brazil (*Melipona interrupta* [126], *M. quadrifasciata anthidioides* [43], *M. seminigra* [126], *M. scutellaris* [139], *M. subnitida* [122], and *Tetragonula (Trigona) spinipes* [88] bees), Indonesia (*Tetragonula aff. biroi* [42], *T. sapiens* [160], and *T. incisa* [136] bees), Malaysia (*Heterotrigona itama* [149] bee), Philippines (*Tetragonula biroi* [137] bee), Thailand (*Tetragonula laeviceps* [138], *T. pagdeni* [151], and *Tetrigona melanoleuca* [138] bees), Vietnam (*Lisotrigona cacciae* [157], *L. furva* [153], and *Tetragonula minor* [146,152] bees), and Australia (*Tetragonula carbonaria* [131,140] bee) have published their SBP studies (Figure 5D). Vietnam is leading the numbers of compounds isolated from SBP, with 34 compounds, followed by Brazil (29 compounds) and Thailand (19 compounds). Australia is the only representative of Oceania reporting eight compounds identified from SBP. Interestingly, there are no reports of isolated compounds from African SBP, although the extracts of Kenyan SBP *Dactylurina schimidti* [163] and Nigerian SBP *Dactylurina studingeri* [164] were reported to have an antimicrobial activity (Figure 5B).

**Figure 6:**
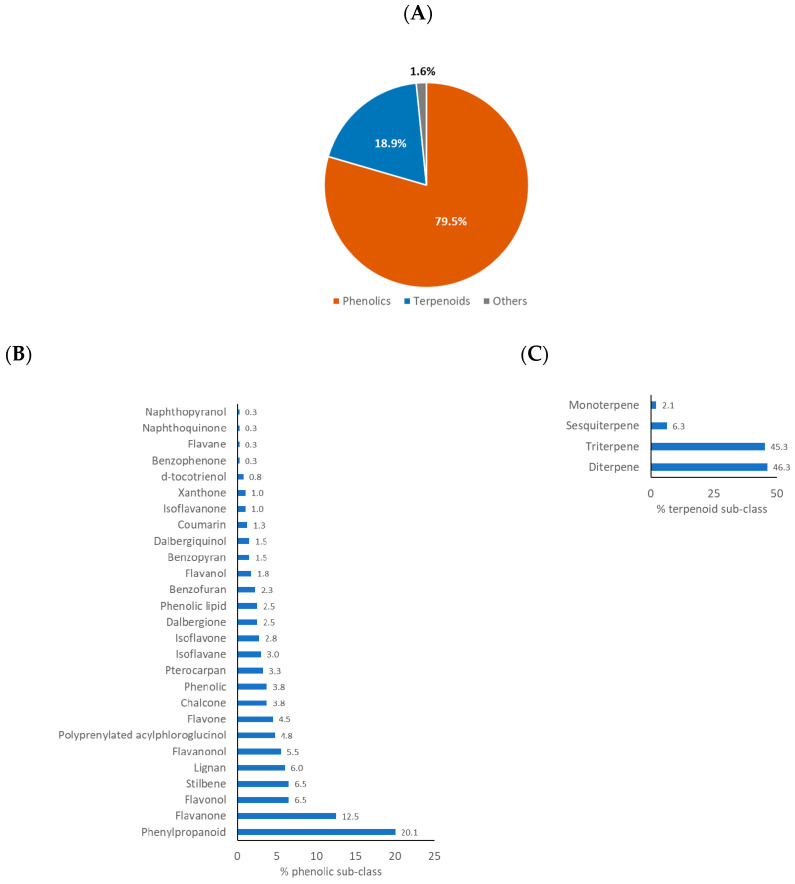
(**A**) Class of compounds isolated from HBP (*n* = 502) (phenolics and terpenoids include their glycosides); (**B**) sub-class of phenolics; (**C**) sub-class of terpenoids (overlapped compounds were removed).

**Figure 7:**
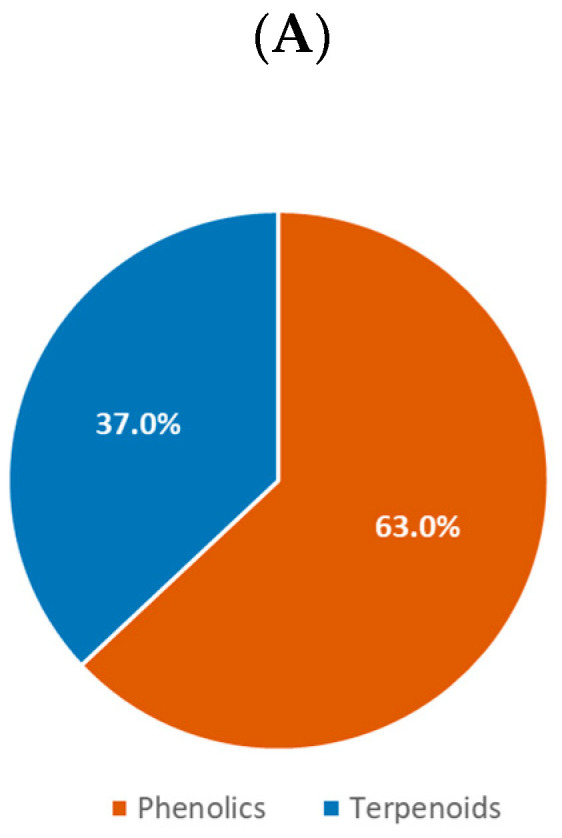

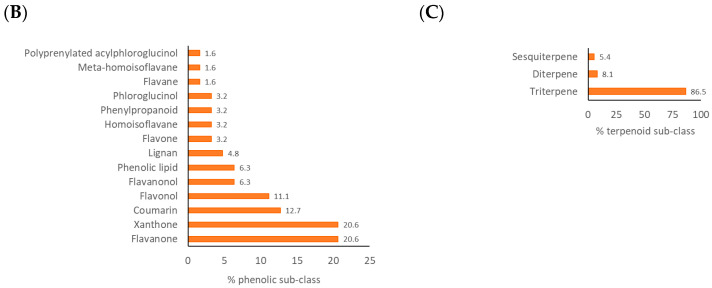
(**A**) Class of compounds isolated from SBP (*n* = 100) (phenolics include their glycosides); (**B**) sub-class of phenolics; (**C**) sub-class of terpenoids (overlapped compounds were removed).

Collation and analysis of the compounds isolated from HBP and SBP revealed that phenolics and terpenoids were the two compound classes that were most often found in propolis. Figure 6A and Figure 7A highlighted that phenolic compounds were dominant, with 79.5% and 63.0% of compounds isolated from HBP and SBP, respectively. Following the ways of the phenolic sub-class classification utilized in previous propolis reviews [20,26,165], nearly 30 sub-classes of phenolics were found in HBP but only half of them were identified in SBP (Figure 6B and Figure 7B). Phenylpropanoids (20.1%) and flavanone (12.5%) were commonly present in HBP (Figure 6B), while flavanone (20.6%) and xanthone (20.6%) were often found in SBP (Figure 7B).

The terpenoids accounted for 18.9% of all compounds found in HBP and 37.0% in SBP (Figure 6C and Figure 7C). They consisted of triterpenoids, diterpenoids, sesquiterpenoids, and monoterpenoids. The HBP diterpenes and triterpenes were similarly represented, with 46.3% and 45.3%, respectively. However, triterpenes occupied the highest proportion of compounds identified in SBP, with 86.5%. Approximately 6.0% of terpenoids identified in both types of propolis were sesquiterpenes. Only two monoterpenes, tschimgin and tschimganin, have been reported so far [107]. These two compounds were isolated from Iranian HBP of which a plant *Ferula* spp. is their botanical source [107]. Interestingly, only 5 out of 578 propolis compounds were identified as glycoside compounds including isorhamnetin-3-*O*-rutinoside from Cretan (Greek) *A. mellifera* HBP [96], ent-8(17)-labden-15-*O*-α-l-rhamnopyranoside, and ent-8(17)-labden-15-*O*-(3′-*O*-acetyl)-α-l-rhamnopyranoside from Salvadorian *A. mellifera* HBP [64], and naringenin-4′-*O*-β-d-glucopyranoside and myricetin-3-*O*-β-d-glucopyranoside from Brazilian *Melipona interrupta* and *M. seminigra* SBP [126].

### 2.3. Characteristic Chemical Class of Propolis

**Figure 8:**
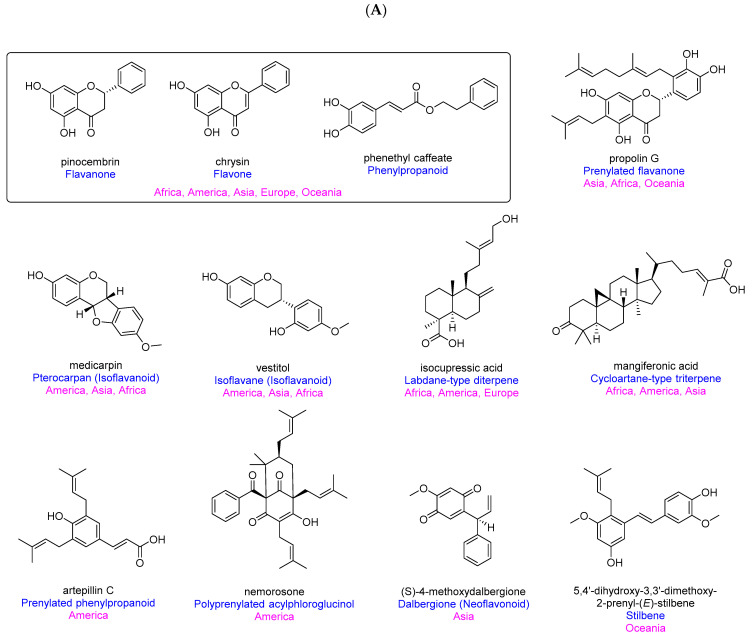

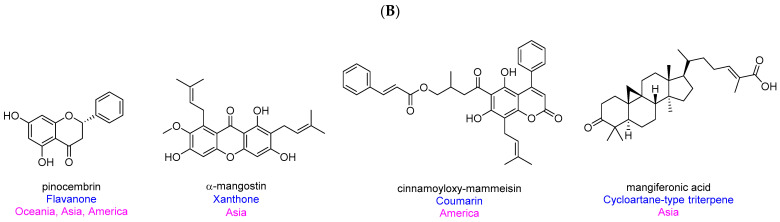
Characteristic chemical constituents of propolis (black: compound name; blue: compound class; purple: continental distribution). (**A**) HBP; (**B**) SBP.

According to the chemo-geographic data, Bankova [165] classified six main HBP types, consisting of (a) Poplar propolis from Europe, North America, and the non-tropical regions of Asia, containing flavones, flavanones, and phenylpropanoids; (b) Birch propolis from Russia containing flavones and flavonols; (c) green propolis from Brazil containing prenylated phenylpropanoids; (d) red propolis from Cuba and Venezuela containing polyprenylated acylphloroglucinols; (e) Pacific propolis from Okinawa and Taiwan containing prenylated flavanones; and (f) Canarian propolis from Canary Islands containing furofuran lignans. More recently, Salatino and his co-workers [26] suggested five HBP types based on climate zones, including (a) temperate poplar propolis derived from *Populus* spp. with flavonoids, esters of phenylpropanoids; (b) Brazilian tropical green propolis with prenylated phenylpropanoids and caffeoylquinic acids; (c) Brazilian tropical brown propolis derived from *Clusia* spp. with polyprenylated acylphloroglucinols; (d) sub-tropical and tropical Pacific propolis derived from *Macaranga* spp., with geranyl flavonoids; and (e) Greek, Cretan, and Turkish propolis (Mediterranean region) with either diterpenoids or anthraquinones. Several reviews of SBP reported the chemical compositions and their biological activities [29,30,166]. However, most of the compounds reviewed were identified by HPLC, GC-MS, and LC-MS. In this review, we only included fully characterized compounds from HBP and SBP and categorized them based on their chemical classes (Figure 8).

Flavanone, flavone and phenylpropanoid, particularly phenylpropanoid esters, are often found from temperate HBP in Africa, America, Asia, Europe, and Oceania (Figure 8A). These compounds were likely foraged from *Populus* spp. (Algeria [124,154], Mexico [101], Uruguay [68], China [120], Bulgaria [45], and the Netherlands [65]), *Zuccagnia punctate* (Argentina [98]), *Liquidambar styraciflua* (Honduras [119]), *Pinus halepensis* (Jordan [113]), *Styrax* spp. (Thailand [123]), *Betula verrucosa* (Russia [25]), or *Xanthorrhoea* spp. (Australia [44]) (Table 1). Pinocembrin, chrysin, and caffeic acid phenyl ester (CAPE or phenethyl caffeate) are three common compounds present in these types of propolis. They showed a wide range of biological activities such as antioxidation, anticancer, antimicrobes, anti-inflammation, neuroprotection, and hepatoprotection (Table 2) [167,168,169].

Prenylated flavanone-type compounds, which were previously classified as a chemical marker of Pacific HBP, have been found not only in Asia (Japan [75,85], Oman [125], and Taiwan [70,84]), and Oceania (Fiji [143] and Solomon Island [106,117,118]), but also in Africa (Egypt [92,100] and Nigeria [141]). These compounds originated from *Macaranga* spp. (predominantly *M. tanarius*) and *Azadirachta indica* (Table 1). A representative of this compound class is propolin G, which has been found to have strong antioxidant, neuroprotective, and hepatoprotective properties (Table 2) [37,170].

Two sub-classes of isoflavanoids, pterocarpan and isoflavane, have been found from HBP in America (Brazil [89], Cuba [81,129], and Mexico [103]), Asia (Nepal [78,86,87]) and Africa (Nigeria [141,154]). *Dalbergia* spp. has been known as a botanical source of these specific propolis (Table 1). Two compounds, medicarpin and vestitol, that were frequently isolated in these HBP, both exhibited antibacterial activity [171,172]. Moreover, medicarpin was found as a potential anticancer and bone healing agent [173,174], while vestitol showed potent antioxidant and anti-inflammatory properties [171,175,176] (Table 2).

Labdane-type diterpene compounds, which were previously classified as major chemical components of Mediterranean HBP, have been found from HBP not only in the Mediterranean area (Greece [96,105], Italy [73], Algeria [124], and Libya [133,161]) but also in America (Brazil [48,53] and Colombia [95]). Botanical sources of these compounds were determined from *Araucaria heterophylla* (Brazil [48]), *Baccharis* spp. (Brazil [53]) and *Cistus* spp (Algeria [124]) (Table 1). The labdane-type diterpenes in propolis, particularly isocupressic acid, showed strong antibacterial and antitrypanosomal activities (Table 2) [48,73,161].

Cycloartane-type triterpenes have been identified from African (Cameroon [130,132], Libya [161] and Nigeria [147]), American (Brazil [67,79,90] and Mexico [158]) and Asian (Indonesia [114], Myanmar [93], and Thailand [148]) HBP. Plant sources of these triterpenes were identified from *Anacardium occidentale* (Brazil [90]), *Bursera simaruba* (Mexico [158]) and *Mangifera indica* (Brazil [79], Indonesia [114], Myanmar [93], and Thailand [148]) (Table 1). Mangiferonic acid, which is a common compound in these propolis, exhibited antidiabetic, antitrypanosomal, and antimalarial properties (Table 2) [37,147,161].

Whilst finding similar components in propolis is relatively common, propolis of different continents also has their characteristic chemical classes. The Brazilian green propolis from *Baccharis* spp. is a source of a prenylated phenylpropanoid, artepillin C, which exhibits a wide spectrum of biological activities including antioxidation, anticancer, antibacteria, antifungi, antitrypanosome, and anti-inflammation (Table 2) [177,178]. The South American brown propolis (mainly in Cuba and Venezuela) from *Clusia* spp. is famous for its high content of polyprenylated acylphloroglucinols. Nemorosone in this propolis showed potent antioxidant, anticancer, antileishmanial, antitrypanosomal, and antiviral properties (Table 2) [66,179]. The Nepalese propolis from *Dalbergia* spp. is characterized by the presence of the open-chain neoflavonoids dalbergione. The compound, 4-methoxydalbergione, and its analogues, are known to contribute to the anticancer and anti-inflammatory activities of this propolis (Table 2) [180]. In Australia, HBP collected in Kangaroo Island, South Australia, is unique with a large number of stilbenes accumulated from the exudates of the Australian native sedge plant *Lepidosperma* spp. [121,145]. The Kangaroo Island propolis displayed four times stronger antioxidant activity than the Brazilian green propolis [116]. The compound, 5,4′-dihydroxy-3,3′-dimethoxy-2-prenyl-(*E*)-stilbene, present in this propolis, inhibited the growth of cancer cell lines more potently than the anticancer agent tamoxifen (Table 2) [145].

With regards to SBP components (Figure 8B), flavanone-rich propolis are common in Asia (Indonesia [42] and Philippines [137]), America (Brazil [88,122]) and Oceania (Australia [131]). In addition to flavanone, Thai [138,151], and Vietnamese [157] SBP are particularly rich in xanthones. Studies indicated *Garcinia mangostana*, which is a common plant in both countries, is a botanical source of these propolis [138,151,157]. A major xanthone component of Thai and Vietnamese SBP, α-mangostin, has antioxidant, anticancer, anti-inflammatory, antibacterial, antimalarial, antiviral, anti-obesity, and neuroprotective activities [181]. One type of Brazilian SBP originating from the plant *Kielmeyera* sp. contained coumarin-type compounds as chemical markers [139]. Cinnamoyloxy-mammeisin present in this Brazilian SBP exhibited anti-inflammatory and antibacterial activities (Table 2) [182,183]. Similarly to honey bees in Brazil [79], Myanmar [93], and Thailand [148], stingless bees in Indonesia [160] and Vietnam [146] also collect resin from *Mangifera indica* to produce propolis containing mainly cycloartane-type triterpenes.

## 3. Physicochemical Property Profiles and Chemical Diversity Analysis of Propolis Components

The chemical space and diversity coverage of HBP and SBP components reviewed in this work were analysed using well-established descriptors and chemoinformatic methods. In order to assess the potential of compounds isolated from HBP and SBP for the development of pharmaceuticals and nutraceuticals based on the chemical structure perspective, the HBP and SBP molecular databases were compared to two public repositories including a large collection of food chemicals (FC) (http://foodb.ca/) and FDA-approved small molecule drugs obtained from Drugbank (DB) [227] (https://www.drugbank.ca/) (Table 3).

**Figure 9:**
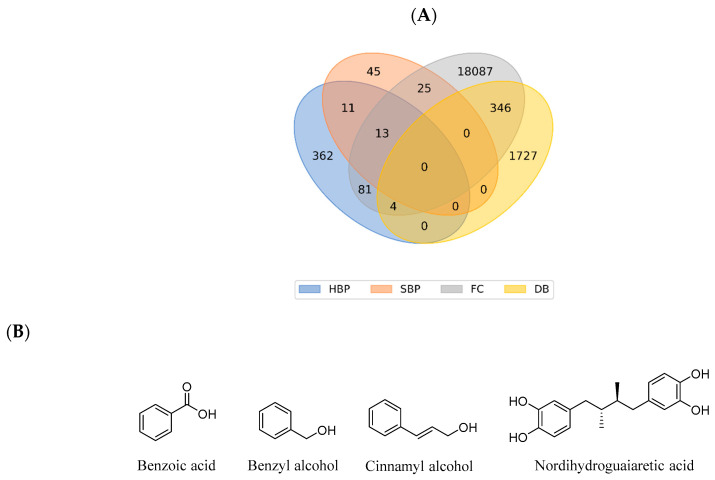
(**A**) Overlapping compounds in four datasets HBP, SBP, FC and DB; (**B**) Chemical structures of the four HBP compounds present in both FC and DB.

Chemoinformatic analysis of the four databases (Figure 9A) indicated that 77% of HBP and 48% of SBP compounds were unique, with 21% of HBP compounds and 40% of SBP compounds being present in the FC database. Of the 24 compounds that were found in both HBP and SBP, 13 compounds were also in the FC database. Four HBP compounds were found in the DB database whereas none of SBP compounds were identified in DB. The four compounds shared between HBP, FC, and DB included a fungistatic agent—benzoic acid [228]; an anaesthetic and antimicrobial agent—benzyl alcohol [228]; a support agent in the diagnosis of allergic contact dermatitis—cinnamyl alcohol [228]; and an antineoplastic agent—nordihydroguaiaretic acid (masoprocol) [228] (Figure 9B).

### 3.1. Physicochemical Property Profiles

**Figure 10:**
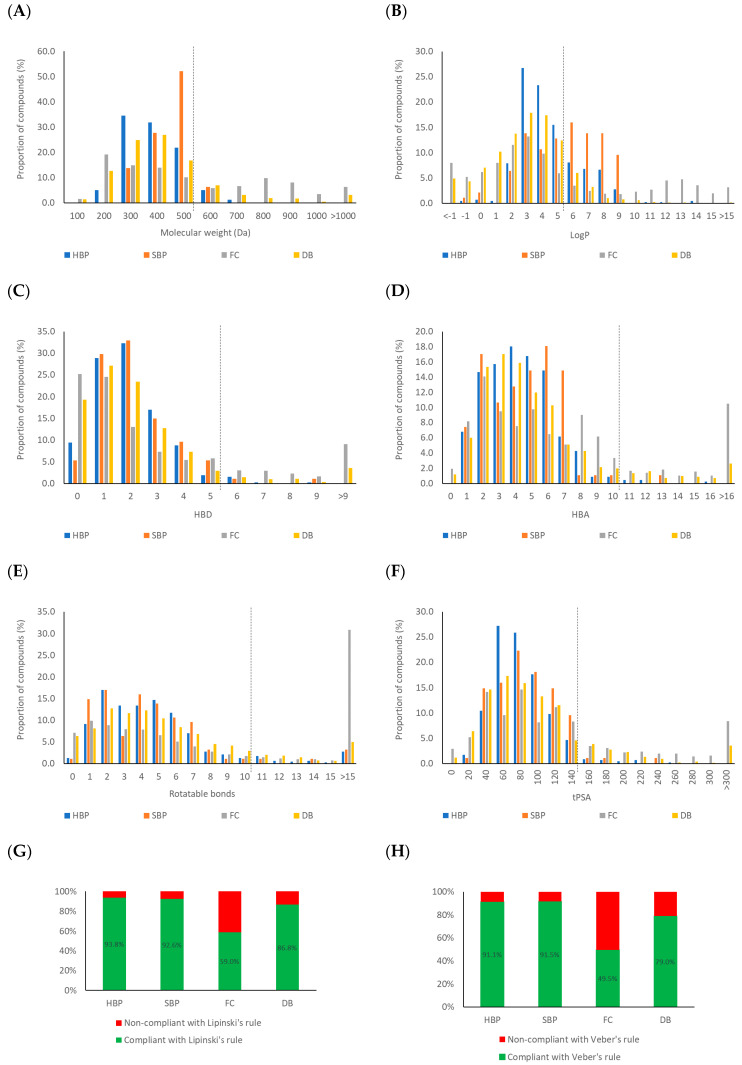
Comparisons of the physicochemical properties (Lipinski and Veber descriptors) of propolis components, food chemicals and approved drugs. (**A**) Molecular weight; (**B**) LogP; (**C**) hydrogen bond donors; (**D**) hydrogen bond acceptors; (**E**) rotatable bonds; (**F**) topological polar surface area; (**G**) Lipinski compliance; and (**H**) Veber compliance.

From the analysis of approximately 2,500 drugs and candidate drugs reaching phase II clinical trials, Lipinski and his co-workers [229] defined four simple physicochemical parameter ranges (molecular weight ≤ 500, logP ≤ 5, hydrogen bond donor (HBD) ≤ 5, and hydrogen bond acceptor (HBA) ≤ 10) as an empirical rule or guide to assess the potential cellular permeability of the molecule. According to Lipinski’s rule, there is a high probability that bioactivity of the molecule via the oral route of administration will be low if it has more than one violation of the four criteria. However, meeting Lipinski’s rule (often referred to as the Rule of Five) is no guarantee that a compound is drug-like [230,231]. By measuring the oral bioavailability of 1100 drug candidates in rats, Veber and co-workers [230] found that the number of rotatable bonds (RB) and topological polar surface area (tPSA) of a molecule link with its oral bioavailability. An RB of 10 or fewer and a tPSA of 140 Å^2^ or less support the oral bioavailability. These two parameters became additional features to assess the oral bioavailability property of potential drug-like molecules [230]. Therefore, the chemical space of the HBP and SBP and two reference databases (FC and DB) was analysed based on the six physicochemical properties (molecular weight, logP, HBD, HBA, rotatable bond, and tPSA) (Figure 10).

The molecular weight profile (Figure 10A) shows both HBP and SBP compounds are in a range from 100 Da up to 700 Da (108.14 Da–709.20 Da for HBP compounds, and 256.26 Da–552.62 Da for SBP compounds). Approximately 94% of HBP and SBP compounds have molecular weights below 500 Da, while 60% of compounds in the FC and 83% in the DB are in this range. Most HBP compounds distribute between 300 Da and 400 Da, while SBP compounds distribute relatively higher from 400 Da to 500 Da. The logP histogram (Figure 10B) shows a logP distribution of HBP compounds ranging from 3 to 5, which is similar to compounds in the FC and DB databases, whereas SBP compounds have logP mainly distributing higher than 5. This result indicates that compounds identified from SBP are less polar than those from HBP. This is consistent with the fact that a relatively large proportion of SBP compounds (37.0%) are terpenoids, as compared to HBP (18.9%). It was found that an increasing number of HBD and HBA hinders the permeability of a compound across a lipid bilayer membrane resulting in the decrease in its oral bioavailability [229]. The distribution of the calculated HBD (Figure 10C) is similar for both HBP and SBP compounds, with HBD being 5 or less. Of the HBP and SBP compounds, 98% are Lipinski-compliant and most of the compounds possess 1–2 HBD. The HBA of HBP compounds range from 3 to 5, while the HBA of SBP compounds reach a maximum at 6 (Figure 10D). Generally, the HBA profile of HBP is relatively close to the HBA profile of the DB compounds but is different to that of SBP compounds and food chemicals. Interestingly, 99% of HBP and SBP compounds have HBA of 10 or less. The rotatable bond profiles of both HBP and SBP compounds (Figure 10E) show a similar pattern to that of compounds in the DB database with approximately 95% of compounds falling within the Veber-compliant rotatable bond region, while 88% and 64% of compounds in the DB and FC databases, respectively, are in this region. The tPSA of HBP compounds peaks between 60–80 Å^2^, whereas tPSA of SBP compounds is higher between 80–100 Å^2^ (Figure 10F). However, 97% of HBP and SBP compounds have a tPSA of 140 Å^2^ or less, which is significantly more than compounds in the FC (74%) and DB (85%) databases.

Overall, approximately 93% of both HBP and SBP components follow the Lipinski’s rule of five, which is significantly greater than compounds of both the FC (59%) and DB (87%) databases (Figure 10G). Taking Veber’s criteria into account (Figure 10H), about 91% of HBP and SBP compounds follow the rule while only 50% of food chemicals and 79% of approved drugs were compliant. This analysis of the physicochemical properties based on Lipinski and Veber descriptors indicates that there is relatively high chance (about 90%) to find drug-like potential compounds with oral bioavailability in propolis sources. When comparing physicochemical properties of HBP and SBP compounds with those of drugs derived from natural products [232], HBP and SBP compounds are close to those of oral, topical and inhalant drugs, and significantly different from injectable drugs (Supporting Information 1, Figure S1).

### 3.2. Structural Diversity

#### 3.2.1. Fingerprint-Based Diversity

**Figure 11:**
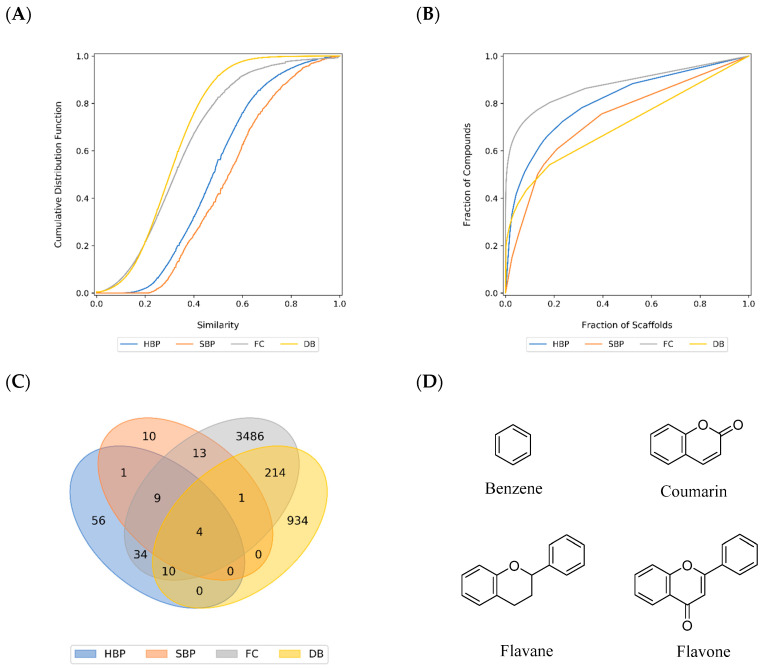
Structural diversity of HBP, SBP and reference compound datasets. (**A**) Fingerprint-based diversity; (**B**) scaffold diversity—CSR curve; (**C**) scaffold overlap; (**D**) four overlapped scaffolds present in all four datasets.

Despite the fact that physicochemical properties represent an intuitive manner to describe compound databases, they do not provide information of the atom connectivity and information of the topology. For instance, it might happen that two molecules with different chemical structures share similar or even identical drug-like properties based on the Lipinski and Veber descriptors. Therefore, in addition to using physicochemical properties to characterize the HBP and SBP, the datasets were further characterized by molecular fingerprints to describe rapidly, but efficiently, the molecular structures based on their atom connectivity and topology, and complement the diversity analysis of compound collections [233]. Molecular diversity analysis based on molecular fingerprint representations provides information on the diversity of the entire molecule by comparing the presence or absence of fragment fingerprint features within the molecule [234]. In this work, the molecular diversity was computed using the well-known fingerprint representation Molecular ACCess System (MACCS) keys (166-bits) and the Tanimoto coefficient [235]. High value of the Tanimoto coefficient (close to one) indicates high structure similarity (based on that particular fingerprint), hence, a low diversity. The cumulative distribution function of the pairwise MACCS keys fingerprints/Tanimoto similarity values for each dataset (Figure 11A) indicated that SBP was less diverse than HBP. The relative order of diversity was further confirmed by the median Tanimoto similarity values (Table 4) with 0.545 for SBP versus 0.479 for HBP. Having the median similarity values of 0.302 and 0.323, DB and FC were the first and second diverse databases, respectively. The results of the fingerprint diversity for the reference collections (FC and DB) are consistent with previous reports [236].

#### 3.2.2. Scaffold Diversity

To further characterize the diversity of compound datasets, molecular scaffolds are commonly used in chemoinformatic analysis as they provide direct information of the molecular structure and are intuitive to interpret [236,237]. A scaffold is defined as the core structure of the compound consisting of all of its rings and connecting linkers [238]. A scaffold with a privileged substructure character associated with specific biological activities can be used as a template for target-directed compound development or compound library design [239]. In this analysis, the scaffold diversity of the four databases was quantified using cyclic system recovery (CSR) curves, which represents a way to capture the distribution of compounds in the cyclic systems of a compound collection [240]. The lower the area under the CSR curve (AUC), the larger the scaffold diversity [241]. The graph (Figure 11B) indicated that the DB database, being the closest to a diagonal, was the most diverse (AUC = 0.707). With an AUC value of 0.737, SBP was the second most diverse dataset, followed by HBP (0.809) and FC (0.878). The high scaffold diversity of the approved drug dataset was expected, not only because of the dataset size but also because of the nature of the molecules (directed to a broad range of molecular targets and therapeutic indications). However, it was remarkable that the SBP dataset had high scaffold diversity regardless its relatively small size (94 molecules). As for the FC dataset, it has been shown that the low scaffold diversity (despite the large size with 18,556 molecules) is due to the high number (32%) of acyclic compounds [236].

The scaffold diversity can also be assessed from the CSR curves by the fraction of cyclic systems required to retrieve 50% (F_50_) of the molecules of the dataset. Thus, larger F_50_ values indicate higher diversity [241]. Based on this metric (Table 4), the diversity of the four databases decreased in the following order: SBP > DB > HBP > FC. In general, both AUC and F_50_ values obtained from CSR curves indicated that SBP had quantitatively higher scaffold diversity than HBP even though SBP displayed less fingerprint-based diversity than HBP.

**Figure 12:**
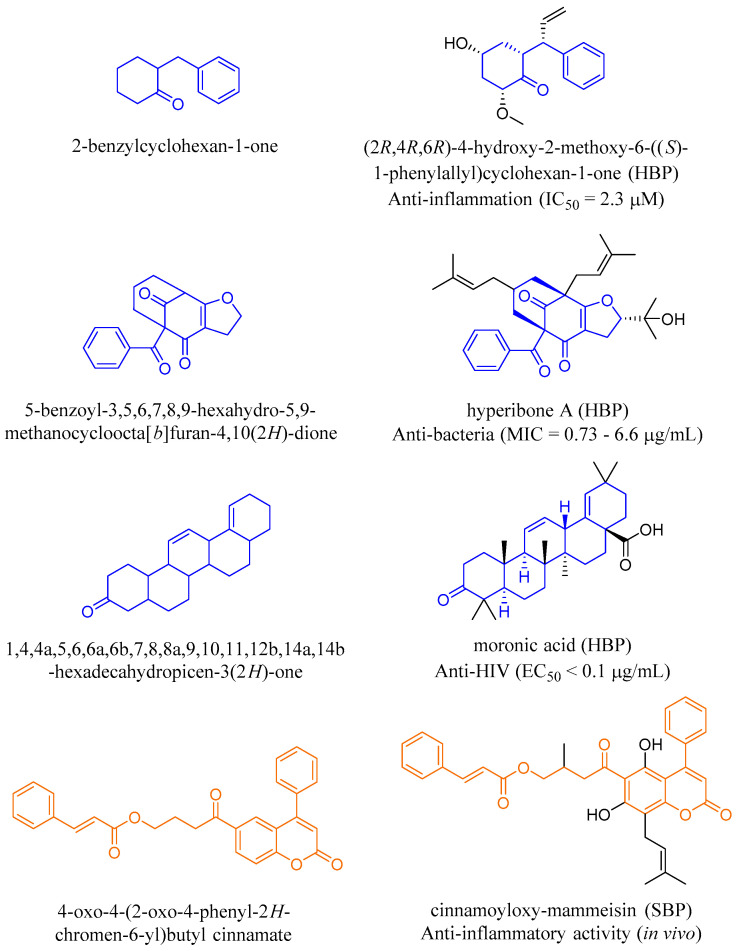
Examples of unique scaffolds and their representative compounds identified in HBP (blue) and SBP (orange).

The comparisons of the scaffolds in HBP and SBP with the scaffolds in the FC and DB databases (Figure 11C) indicated that HBP shared 14 scaffolds with both FC and DB compounds while SBP shared five scaffolds with both FC and DB. Four scaffolds were identified to be present in all four datasets including benzene, coumarin, flavane, and flavone scaffolds (Figure 11D). The analysis also revealed 56 unique scaffolds in 89 compounds of HBP and 10 unique scaffolds in 13 compounds of SBP (Supporting Information 2). Approximately 50% of the unique scaffolds from HBP were found in tropical regions and only 10% were found in temperate areas. In terms of SBP, only one unique scaffold was identified in a sub-tropical area of Australia (South East Queensland) whereas the other nine unique scaffolds were found from tropical regions. Several representative compounds containing the unique scaffolds of HBP and SBP such as (2*R*,4*R*,6*R*)-4-hydroxy-2-methoxy-6-((*S*)-1-phenylallyl)cyclohexan-1-one [78], hyperibone A [91], moronic acid [61], and cinnamoyloxy-mammeisin [182] exhibited potent anti-bacterial, anti-HIV, and anti-inflammatory activities (Figure 12). About 30% of unique HBP scaffolds and 60% of unique SBP scaffolds have not been assessed for their biological activities.

## 4. Conclusions and Perspectives

It is generally accepted that the chemistry of propolis depends on the bee species and the flora of the region inhabited by the bees. However, this study has shown that bees in different regions harvest similar compounds from different plant families, such as chrysin, pinocembrin, mangiferonic acid, and isocupressic acid. We also found that both honey bees and stingless bees are attracted by similar flavanone and cycloartane-type triterpenes. Although the current literature does not identify the mechanisms that drive bees to recognize the compounds, the coincidences in chemical components of propolis indicate that bees actively and selectively forage plant resins containing bioactive compounds, particularly antimicrobial compounds (antibacterial, antifungal, antiparasitic, and antiviral properties), to protect themselves against pathogens and predators.

A unique compound database consisting of 502 compounds from HBP and 100 compounds from SBP (of which 24 compounds overlapped between the two) was assembled in this work and is freely accessible in the Supporting Information. Although HBP and SBP components are mainly phenolics and terpenoids originally from plant resins, new and novel compounds in propolis continue being identified. This study showed that over 90% of the compounds found from HBP and SBP have oral bioavailability property and fit in the chemical space of drug-like molecules as defined by Lipinski’s and Veber’s rules [229,230], which is a greater proportion than is observed in the food chemical and approved drug databases.

Chemical diversity analysis provided quantitative evidence that HBP had higher structural diversity based on molecular fingerprints, but lower scaffold diversity than SBP. However, the larger number of HBP compounds, as compared to SBP compounds (502 compounds versus 100 compounds), could significantly affect the structural diversity analysis. Therefore, we may find that the SBP database has higher structural diversity when additional SBP compounds are discovered. Despite the relatively small number of compounds identified from HBP and SBP, they have provided access to 66 novel scaffolds, which are not currently represented in food chemicals and approved drugs. Interestingly, 31 novel scaffolds from HBP and 9 novel scaffolds from SBP were from the compounds identified in tropical regions where bees can access a wide range of floral sources, due to the high biodiversity in the tropical zone. Although we remain largely unaware of their therapeutic benefits, research has revealed that over 50% of compounds containing these unique scaffolds showed at least one biological activity including anti-microbial, anti-inflammatory, and anticancer properties. The identification of these novel scaffolds may be valuable starting points for future drug design and development to treat infectious and chronic diseases.

## Figures and Tables

**Table 1:** Botanical sources of propolis categorized by chemical class.

Plant Species	Plant Family	Characteristic Chemical Class	Bee Species	Country
*Acacia paradoxa*	Fabaceae	Chalcone Flavanonol	*A. mellifera*	Australia [121]
*Anacardium occidentale*	Anacardiaceae	Cycloartane-type triterpene	*A. mellifera*	Brazil [90]
*Araucaria heterophylla*	Araucariaceae	Labdane-type diterpene	*A. mellifera*	Brazil [48]
*Azadirachta indica*	Meliaceae	Prenylated flavanone	*A. mellifera*	Oman [125]
*Baccharis* spp.	Asteraceae	Flavanone/Flavanonol Flavone/Flavonol Phenylpropanoid ester Prenylated phenylpropanoid Labdane-type diterpene	*A. mellifera*	Brazil [53,59]
*Betula verrucosa*	Betulaceae	Flavone/Flavonol	*A. mellifera*	Russia [25]
*Bursera simaruba*	Burseraceae	Cycloartane-type triterpene	*A. mellifera*	Mexico [158]
*Cistus* spp.	Cistaceae	Labdane-type diterpene	*A. mellifera*	Algeria [124]
*Clusia* spp.	Clusiaceae	Polyprenylated acylphloroglucinol	*A. mellifera*	Cuba [66] and Venezuela [77]
*Corymbia torelliana*	Myrtaceae	Flavanone/Flavanonol	*T. carbonaria*	Australia [131]
*Dalbergia* spp.	Fabaceae	Pterocarpan Isoflavone Isoflavane Dalbergione	*A. mellifera*	Brazil [89], Cuba [81,129], Mexico [103], Nepal [78,86,87], and Nigeria [141,154]
*Garcinia mangostana*	Guttiferae	Xanthone	*T. laeviceps* *T. pagdeni* *L. cacciae*	Thai [138,151] and Vietnamese [157]
*Kielmeyera* sp.	Calophyllaceae	Coumarin	*M. scutellaris*	Brazil [139]
*Lepidosperma* spp.	Cyperaceae	Stilbene	*A. mellifera*	Australia [121,145]
*Liquidambar styraciflua*	Altingiaceae	Flavanone Phenylpropanoid ester	*A. mellifera*	Honduras [119]
*Macaranga* spp.	Euphorbiaceae	Prenylated flavanone	*A. mellifera*	Japan [75,85], Taiwan [70,84], Fiji [143], Solomon Island [106,117,118], Egypt [92,100] and Nigeria [141]
*Mangifera indica*	Anacardiaceae	Cycloartane-type triterpene	*A. mellifera* *Tetragonula sapiens* *T. minor*	Brazil [79], Indonesia [114,160], Myanmar [93], Thailand [148], Vietnam [146]
*Pinus halepensis*	Pinaceae	Flavanone/Flavanonol Flavone/Flavonol	*A. mellifera*	Jordan [113]
*Populus* spp.	Salicaceae	Flavanone/Flavone Phenylpropanoid ester	*A. mellifera*	Algeria [124,154], Mexico [101], Uruguay [68], China [120], Bulgaria [45], Netherland [65]
*Styrax* spp.	Styracaceae	Flavanone/Flavanonol Flavone/Flavonol Phenylpropanoid ester	*A. mellifera*	Thailand [123]
*Xanthorrhoea* spp.	Xanthorrhoeaceae	Flavanone	*A. mellifera*	Australia [44]
*Zuccagnia punctate*	Caesalpinieae	Flavanone/Flavonol	*A. mellifera*	Argentina [98]

**Table 2:** Representative compounds in propolis with known biological activities.

Compound	Chemical Class	Phenotypic Activity	Molecular Target Activity
Artepillin C	Prenylated phenylpropanoids	Antibacteria (inhibition of *B. cereus, B. Subtilis, M. lysodeikticus, P. aeruginosa, E. aerogenes, M. smegmatis, S. faecalis, E. coli, C. equi*, and *S. aureus* [177]) Antifungi (inhibition of *C. albicans, C. tropicalis, C. neoformans, S. cerevisiae, A. fumigatus, A. flavus, A. niger, M.canis, M. gypseum, E. floccosum, T. rubrum,* and *T. mentagrophytes* [177]) Antitrypanosome (inhibition of trypomastigote forms of *T. cruzi* [184]) Antioxidation (in vivo inhibition of lipid peroxidation [185]) Anticancer (inhibition of human cancer cell lines [186,187,188])	Anti-inflammation (in vitro and in vivo inhibition of NO through NF-κB [178])
Caffeic acid phenyl ester—CAPE (Phenethyl caffeate)	Phenylpropanoid ester	Antibacteria (inhibition of *S. aureus, B. subtilis, and P. aeruginosa* [189]) Antivirus (inhibition of AH1N1 [189] and hepatitis C virus [190])	Antioxidation (inhibition of 5-lipoxygenase [191]) Antivirus (inhibition of HIV-1 integrase [192]) Anti-inflammation (in vivo inhibition of COX- 2 [193], inhibition of NF-κB [194], in vitro and in vivo scavenging of NO and modulation of iNOS expression [195]) Anticancer (inhibition of protein kinase C [196], in vitro and in vivo inhibition of MMP-2, MMP-9 and VEGF [197]) Neuroprotection (scavenging ROS [198]) Hepatoprotection (in vivo inhibition of CYP2E1 [199])
Chrysin	Flavone	Neuroprotection (in vitro and in vivo inhibition of acrylamide-induced toxicity [200]) Antivirus (inhibition of enterovirus 71 [201])	Anticancer (in vitro and in vivo activation of Notch1 signalling [202], regulating MMP-10 and epithelial-mesenchymal transition [203], inhibition of HIF-1a [204]) Anti-inflammation (in vivo inhibition of COX-2 and iNOS [205]) Neuroprotection (inhibition of NF-κB and iNOS [206]) Antidiabetes (inhibition of AGE-RAGE mediated oxidative stress and inflammation [207])
Cinnamoyloxy-mammeisin	Coumarin	Antibacteria (inhibition of methicillin-resistant *S. aureus* adherence to host cells and disruption of biofilm development [183]) Toxicity (low acute toxicity on *Gallleria mellonella* larvae model [183])	Anti-inflammation (in vivo reduction of neutrophil migration by inhibiting the release of TNF-α and CXCL2/MIP-2 associated with inhibition of ERK 1/2, JNK, and p38 MAPK phosphorylation, AP-1, and NF-κB [182])
5,4′-Dihydroxy-3,3′-dimethoxy-2-prenyl-(*E*)-stilbene	Stilbene	Antioxidation (scavenging DPPH radical [116]) Anticancer (inhibiting the growth of NCI-60 cancer cell lines growth [145])	
Isocupressic acid	Diterpene	Antibacteria (inhibition of *S. aureus* [48,73]) Antitrypanosome (inhibition of *T. brucei* [161])	
Mangiferonic acid	Triterpene	Antitrypanosome (inhibition of *T. brucei* [147,161]) Antimalaria (inhibition of *P. falciparum* [161])	Antidiabetes (in vitro inhibition of α-glucosidase [208])
α-Mangostin	Xanthone	Antibacteria (inhibition of *S. epidermidis* [209], and *S. aureus* biofilm formation [210]) Antimalaria (inhibition of *P. falciparum* [211]) Antivirus (inhibition of severe dengue virus [212])	Anticancer (inhibition of fatty acid synthase [213], PERK [214]) Anti-inflammation (inhibition of p65 acetylation, COX-2 and iNOS [215]) Neuroprotection (inhibition of self-induced β-amyloid aggregation [216]) Anti-obesity (inhibition of PPARγ [217])
Medicarpin	Pterocarpan	Antibacteria (inhibition of *P. aeruginosa* and *B. cereus* [172]) Antifungi (inhibition of *T. versicolor* [218])	Bone healing (in vivo bone generation by activating Wnt and notch signalling in pre-osteoblasts [174], in vitro downregulation of GRP78 [219]) Anticancer (Sensitizing human myeloid leukemia cells to TRAIL-induced apoptosis [220], enhancing cytotoxicity of chemotherapy drugs by modulating P-gp-mediated efflux [221])
(*S*)-4-Methoxydalbergione	Dalbergione (Neoflavonoid)		Anti-inflammation (inhibition of the release of β-glucuronidase and superoxide formation induced by phorbol myristate acetate [180]) Anticancer (in vitro and in vivo suppression of osteosarcoma cells through downregulation of JAK2/STAT3 pathway [180])
Nemorosone	Polyprenylated acylphloroglucinol	Antioxidation (scavenging DPPH radical [66]) Anticancer (inhibition of cancer cell lines [66]) Antibacteria (inhibition of *P. larvae, P. alvei* and *S. aureus* [222,223]) Antimalaria (inhibition of *P. falciparum* [223]) Antitrypanosome (inhibition of *T. brucei* and *T. cruzi* [223]) Antileishmania (inhibition of *L. amazonensis* and *L. infantum* [223])	Anticancer (activation of p300 histone acetyltransferase [224])
Pinocembrin	Flavanone	Antibacteria (inhibition of *S. aureus* [225]) Antimalaria (inhibition of *P. berghei* [226])	Neuroprotection (inhibition of MAPK, IκB, NF-κB p65 [167]) Anti-inflammation (inhibition of Th2 cytokines, IL-4, IL-5, IL-13, IκBα, NF-κB p65 phosphorylation, MMP-1, MMP-3, and MMP-13 [167]) Hepatoprotection (inhibition of ROS, PI3K/Akt and SMAD [167])
Propolin G	Prenylated flavanone	Antioxidation (scavenging DPPH radical) [84]	Hepatoprotection (disruption of TGF-β-Smad2/3 signalling by reducing Smad2/3 formation) [170] Neuroprotection (prevention of neuronal death against oxidative stress challenges) [84]
Vestitol	Isoflavane	Antibacteria (inhibition of *S. aureus, S. mutans, S. sobrinus and A. naeslundii* growth) [171,175] Anti-inflammation (in vivo inhibition of neutrophil migration) [171]	

**Table 3:** Summary of the datasets used for comparison.

Dataset	Initial Compounds	Unique Compounds ^b^	Source
HBP	502 ^a^	471	This review
SBP	100 ^a^	94	This review
FC	28,771	18,556	http://foodb.ca/
DB	2413	2077	https://www.drugbank.ca/

^a^ Overlapped compounds were removed. ^b^ Compounds were obtained after being filtered with criteria defined in Supporting Information 1.

**Table 4:** Summary of structural diversity of HBP, SBP, and reference datasets.

Dataset	Size	Chemotype	Median Similarity	Scaffold Diversity (AUC)	Scaffold Diversity (F_50_)
HBP	471	115	0.479	0.809	0.078
SBP	94	38	0.545	0.737	0.158
FC		3772	0.323	0.878	0.004
DB	2077	1164	0.302	0.707	0.144

## Supplementary Materials

Supplementary materials can be found at https://www.mdpi.com/1422-0067/21/14/4988/s1.

## Abbreviations

AGE	Advanced glycation endproducts
AH1N1	Influenza A virus subtype H1N1
AP-1	Activator protein 1
AUC	Area under the cyclic system recovery curve
CAPE	Caffeic acid phenyl ester
COX-2	Cyclooxygenase-2
CSR	Cyclic system recovery
CXCL2	Chemokine ligand 2
CYP2E1	Cytochrome P450 Family 2 Subfamily E Member 1
DB	Drug bank
DPPH	2,2-Diphenyl-1-picrylhydrazyl
ERK 1/2	Extracellular signal-regulated protein kinase 1/2
FC	Food chemicals
FDA	Food and Drug Administration
GC	Gas chromatography
GC-MS	Gas chromatography—Mass spectrometry
GRP78	Glucose Regulated Protein 78
HBA	Hydrogen bond acceptor
HBD	Hydrogen bond donor
HBP	Honey bee propolis
HIF-1a	Hypoxia-inducible factor 1-alpha
HIV-1	Human immunodeficiency virus 1
HPLC	High-performance liquid chromatography
IL-4	Interleukin 4
IL-5	Interleukin 5
IL-13	Interleukin 13
iNOS	Inducible nitric oxide synthase
JAK2	Janus Kinase 2
JNK	Jun N-terminal kinases
LC-MS	Liquid chromatography—Mass spectrometry
LogP	Partition coefficient between octanol and water
MACCS	Molecular ACCess System
MIP-2	Macrophage inflammatory protein 2
MMP-1	Matrix metalloproteinase-1
MMP-2	Matrix metalloproteinase-2
MMP-3	Matrix metalloproteinase-3
MMP-9	Matrix metalloproteinase-9
MMP-10	Matrix metalloproteinase-10
MMP-13	Matrix metalloproteinase-13
MS	Mass spectrometry
NF-κB	Nuclear factor kappa B
NMR	Nuclear magnetic resonance
NO	Nitric oxide
p38 MAPK	p38 mitogen-activated protein kinases
PERK	Protein kinase RNA-like endoplasmic reticulum kinase
PI3K	Phosphoinositide 3-kinases
P-gp	Permeability glycoprotein
PPARγ	Peroxisome proliferator-activated receptor gamma
RAGE	Receptor for advanced glycation endproducts
RB	Rotatable bond
ROS	Reactive oxygen species
SBP	Stingless bee propolis
STAT3	Signal transducer and activator of transcription 3
TGF-β	Transforming growth factor beta
TNF-α	Tumor necrosis factor alpha
tPSA	Topological polar surface area
TRAIL	Tumor necrosis factor-related apoptosis-inducing ligand
VEGF	Vascular endothelial growth factor
Wnt	Wingless-related integration site
