# Supplementary material for: Lessons from Exploring Chemical Space and Chemical Diversity of Propolis Components
Source: Int J Mol Sci. 2020 Jul 15;21(14):4988. doi: 10.3390/ijms21144988 (PMC7404124; doi:10.3390/ijms21144988)
Supplement: Supplementary file 1 [file ijms-21-04988-s001.zip › Supporting information 1_TTran.docx]

Lessons from Exploring Chemical Space and Chemical Diversity of Propolis Components

Trong D. Tran ^1,^*, Steven M. Ogbourne ^1^, Peter R. Brooks ^1^, Norberto Sánchez-Cruz ^2^, José L. Medina-Franco ^2^ and Ronald J. Quinn ^3^

^1^ Genecology Research Centre, School of Science and Engineering, University of the Sunshine Coast, Maroochydore DC, Queensland 4558, Australia; ttran1@usc.edu.au

^2^ Department of Pharmacy, School of Chemistry, Universidad Nacional Autónoma de México, Mexico City 04510, Mexico; medinajl@unam.mx

^3^ Griffith Institute for Drug Discovery, Griffith University, Brisbane, Queensland 4111, Australia; [r.quinn@griffith.edu.au](mailto:r.quinn@griffith.edu.au)

***** Correspondence: ttran1@usc.edu.au; Tel.: +61-7-5459-4579

**1. Experimental Section**

**1.1 Data curation**

The four datasets (HBP, SBP, FC, and DB) were curated using the same procedure. Compounds consisting of multiple components (e.g. salts) were split and the largest component (defined by the number of heavy atoms) was retained, as long as the number of heavy atoms of the second-largest component was less than 70% of the largest component. If this was not the case, the compounds were removed from the dataset, unless the two largest components were identical. Single component compounds as well as the retained component of multiple component compounds were neutralized. Compounds consisting of any element other than H, B, C, N, O, F, Si, P, S, Cl, Se, Br and I, as well as compounds with valence errors, were removed from the dataset. Finally, canonical tautomers of each molecule were generated according to MolVS rules and duplicate structures in the context of each database were removed. For the final 21,198 single component compounds, Canonical simplified molecular-input line-entry system (SMILES) were generated as molecular representation. For this analysis we decided to ignore stereochemistry because about 60 % of the compounds have missing information, mostly in FC and DB datasets but also 127 and 20 compounds for HBP and SBP, respectively. The entire process was performed by using the open-source cheminformatics toolkit RDKit (Version 2019.09.1).

**1.2 Global diversity**

A global diversity analysis was performed over the four datasets considering four criteria

- Molecular fingerprints: The median of the lower triangle from the pairwise similarity matrix computed as the Tanimoto coefficient of MACCS keys (166-bits) fingerprint.
- Molecular scaffolds: area under the cyclic system recovery curve (AUC) and the fraction of chemotypes required to retrieve 50% of the molecules (F50).
- Physicochemical properties: the mean distance of the lower triangle of the pairwise distance matrix computed as the Euclidean distance of six molecular properties scaled, being averaged molecular weight (AMW) partition coefficient octanol/water (SlogP), number of hydrogen bond donors (HBD), number of hydrogen bond acceptors (HBA), number of rotatable bonds (RB), and topological polar surface area (tPSA).
- Size: Number of unique compounds.

**Figure S1.** Comparisons of physicochemical properties of HBP and SBP components, and drugs derived from natural products

| **A)**  **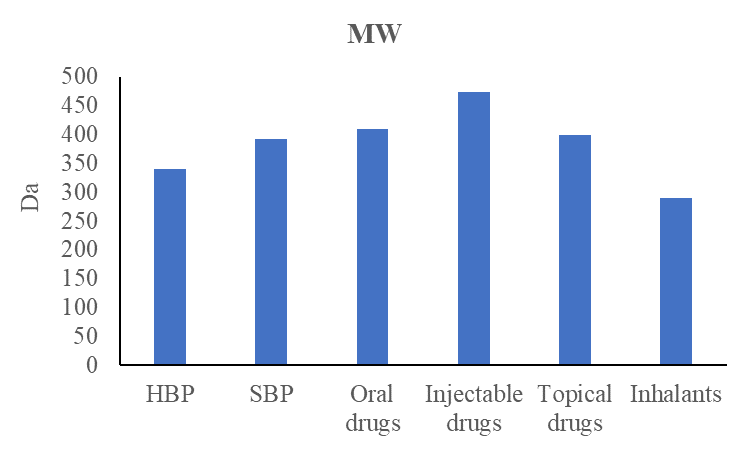** | **B)**  **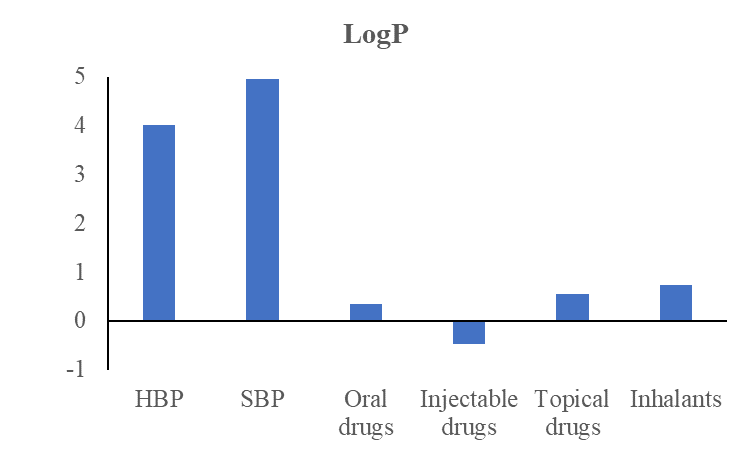** |
| --- | --- |
| **C)**  **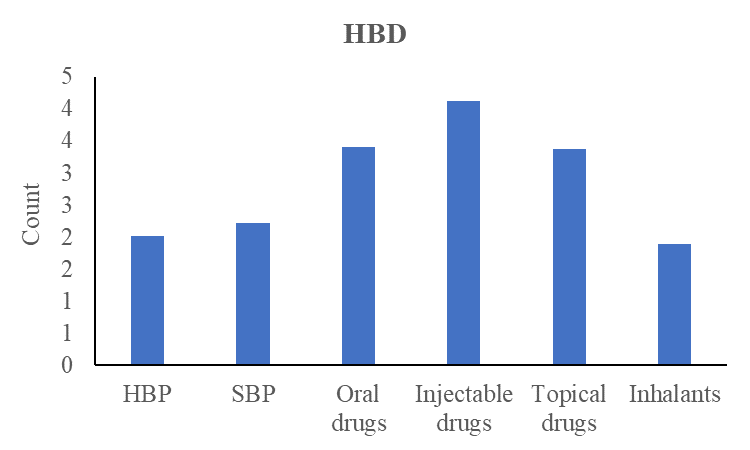** | **D)**  **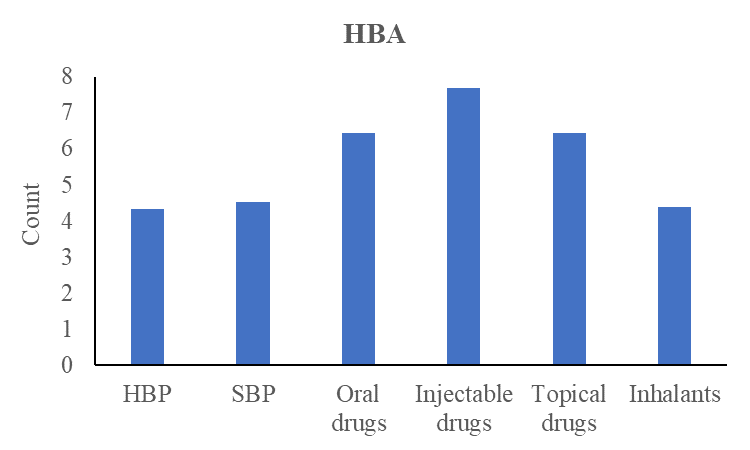** |
| **E)**  **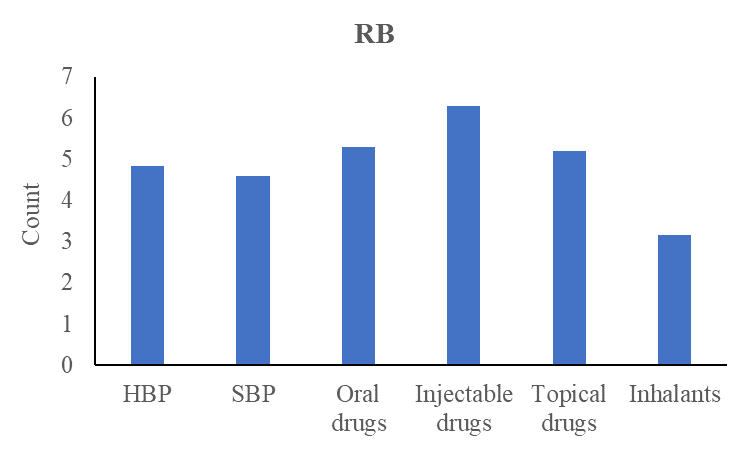** | **D)**  **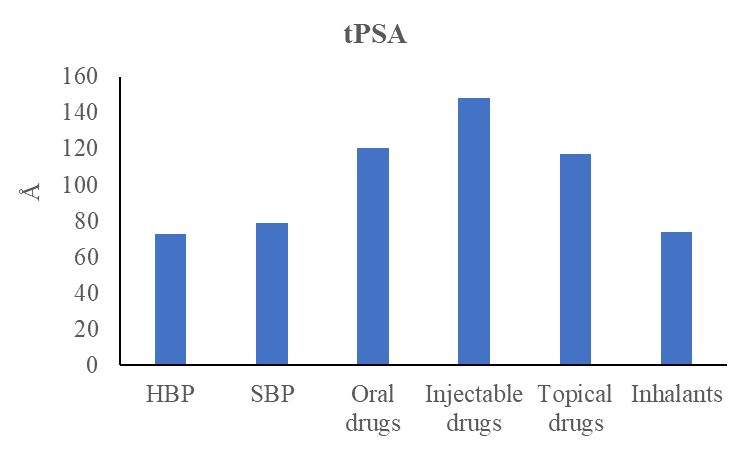** |
